# Association of Arrhythmia in Patients with Cervical Spondylosis: A Nationwide Population-Based Cohort Study

**DOI:** 10.3390/jcm7090236

**Published:** 2018-08-23

**Authors:** Shih-Yi Lin, Wu-Huei Hsu, Cheng-Chieh Lin, Cheng-Li Lin, Chun-Hao Tsai, Chih-Hsueh Lin, Der-Cherng Chen, Tsung-Chih Lin, Chung-Y. Hsu, Chia-Hung Kao

**Affiliations:** 1 Graduate Institute of Biomedical Sciences and School of Medicine, College of Medicine, China Medical University, No. 2, Yuh-Der Road, Taichung 404, Taiwan; oasisbestonly@yahoo.com.tw (S.-Y.L.); Hsuwh@mail.cmuh.org.tw (W.-H.H.); cclin@mail.cmuh.org.tw (C.-C.L.); D7940@mail.cmuh.org.tw (C.-H.T.); d5496@mail.cmuh.org.tw (C.-H.L.); hsuc@mail.cmuh.org.tw (C.-Y.H.); 2 Division of Nephrology and Kidney Institute, China Medical University Hospital, Taichung 404, Taiwan; 3 Division of Pulmonary and Critical Care Medicine, China Medical University Hospital and China Medical University, Taichung 404, Taiwan; 4 Department of Family Medicine, China Medical University Hospital, Taichung 404, Taiwan; 5 Management Office for Health Data, China Medical University Hospital, Taichung 404, Taiwan; orangechengli@gmail.com; 6 College of Medicine, China Medical University, Taichung 404, Taiwan; 7 Department of Orthopedics, China Medical University Hospital, Taichung 404, Taiwan; D16240@mail.cmuh.org.tw; 8 Department of Orthopedics, St. Martin De Porres Hospital, Chiayi 600, Taiwan; zunlin999@gmail.com; 9 Department of Nuclear Medicine, China Medical University Hospital, Taichung 404, Taiwan; 10 Department of Bioinformatics and Medical Engineering, Asia University, Taichung 413, Taiwan

**Keywords:** cervical spondylosis, arrhythmia, population cohort study

## Abstract

Background: Sympathetic activity, including cervical ganglia, is involved in the development of cardiac arrhythmias. Objective: The present study investigated the association between cervical spondylosis and arrhythmia, which has never been reported before. Methods: Patients newly diagnosed with cervical spondylosis (CS) with an index date between 2000 and 2011 were identified from the National Health Insurance Research Database. We performed a 1:1 case-control matched analysis. Cases were matched to controls according to their estimated propensity scores, based on demographics and existing risk factors. Cox proportional hazard models were applied to assess the association between CS and arrhythmia. Results: The CS cohort comprised 22,236 patients (males, 42.6%; mean age, 54.4 years) and non-CS cohort comprised 22,236 matched controls. There were 1441 events of arrhythmia in CS cohort and 537 events of arrhythmia in non-CS cohort, which 252 and 127 events of atrial fibrillation in CS and non-CS cohort, 33 and 12 events of ventricular tachycardia in CS cohort and non-CS cohort, 78 and 35 events of supraventricular tachycardia in CS cohort and non-CS cohort. The CS cohort had an arrhythmia incidence of 11.1 per 1000 person-years and a higher risk [adjusted hazard ratio (aHR) = 3.10, 95% confidence interval (CI) = 2.80–3.42] of arrhythmia, 2.54-fold aHR of ventricular tachycardia (95% CI = 1.70–3.79), and 2.22-fold aHR of atrial fibrillation (95% CI = 1.79–2.76) compared with non-CS cohort. Conclusions: Cervical spondylosis is associated with a higher risk of arrhythmia.

## 1. Introduction

Arrhythmia is a potentially life-threatening condition; it is defined as an irregular heartbeat or abnormal heart rhythm [1]. Cardiac arrhythmia occurs in 11%–58% of the population, of which the most common type is atrial fibrillation [2]. The major determinant of prevalence of arrhythmia is aging of the population [2]. The etiology of cardiac arrhythmia includes abnormalities of the sinoatrial node, atrioventricular node, His-Purkinje system, myocardial remodeling, ionic imbalances, dysfunctional ion channels, and clinical presentations range from asymptomatic to sudden cardiac death [3]. The mechanism of normal aging for arrhythmia is associated with the apoptosis and subsequent fibrofatty infiltration of the myocardial cells, which affects the neighboring conduction system [4]. In addition to normal aging, studies have reported that coronary artery disease [5], air pollution [6], and alteration of the autonomic nervous system [7] (ANS), age, gender, smoking, and races [8], are risk markers of cardiac arrhythmia.

Franciosi et al. found that alterations in levels of neuronal growth factors during ANS development can lead to cardiac arrhythmias [9]. Furthermore, Shen et al. determined that the ANS mechanism involved in triggering arrhythmia could be arrhythmogenic or antiarrhythmic: sympathetic activity and parasympathetic activity are triggers for atrial fibrillation whereas sympathetic activation and parasympathetic activation are arrhythmogenic and antiarrhythmic for ventricular fibrillation, respectively [10].

The cervical spine harbors the cervical ganglia, which are the paravertebral ganglia of the sympathetic nervous system [10]. The sympathetic nerves of the right heart are predominantly from the right middle cervical ganglion and the sympathetic nerves of left heart is predominantly from the left middle cervical ganglion, while the remaining sympathetic innervation is from satellite ganglion, the distribution of which is more prominent in the left heart than right heart [10]. Cardiac parasympathetic innervation is through the vagusnerve [10].

Previous research into the effects of ANS alterations has focused on triggers of sympathetic neuronal activity and blocking agents, and most of these studies have used animal models [11,12,13]. Degenerative disease of cervical spine, naming cervical spondylosis (CS), might cause compression of spinal canal and irritation of ANS [14]. Cervical spondylosis is prevalent among people older than 50 years [14]. Most common clinical signs of CS are pain and motor function impairment [14]. Peng et al. have described two cases of cervical spondylosis that had secondary hypertension and successfully treated the two cases with anterior cervical discectomy and fusion [15]. To our knowledge, no clinical research has investigated whether spondylosis of the spine is associated with a risk of arrhythmia, and the association between CS and arrhythmia is thus unknown.

This study, therefore, used the National Health Insurance Research Database (NHIRD) [15], which represents the Taiwanese population, to test the hypothesis that patients with CS have an increased risk of arrhythmia.

## 2. Methods

### 2.1. Data Source

A population-based retrospective cohort study was conducted using the Longitudinal Health Insurance Database 2000 (LHID2000) derived from the NHIRD of the Taiwan National Health Insurance (NHI) program. The Taiwan NHI program has covered 99% of the Taiwan population (including 23.74 million) since 1995 and is thus a thorough and representative sample of the population [16]. Details of the NHI program and LHID2000 have been well documented [17,18].

### 2.2. Ethics Statement

The NHIRD encrypts patient personal information to protect privacy and provides researchers with anonymous identification numbers associated with relevant claims information, including sex, date of birth, medical services received, and prescriptions. Therefore, patient consent is not required to access the NHIRD. This study was approved to fulfill the condition for exemption by the Institutional Review Board (IRB) of China Medical University (CMUH-104-REC2-115-CR2). The IRB also specifically waived the consent requirement.

### 2.3. Participants

Patients aged 18 years and older who had been diagnosed with CS without myelopathy (ICD-9-CM codes 721.0) and CS with myelopathy (ICD-9-CM codes 721.1) between 2000 and 2010 were selected as the CS cohort (the case group). CS patients were considered while they had received medical care at least three times outpatient visits and/or one-time hospitalizations, for principal/secondary diagnosis of CS in 2000–2010. Further, the diagnosis of CS must be absent during 1996–2000, thus diagnosis of CS would be first diagnosed between 2000–2010. The accuracy of diagnosis of cervical spondylosis registered in NHIRD has been reported and validated [19]. The index date was the date of CS diagnosis.

To reduce selection bias of this observational cohort studies, we used a 1:1 case-control matching analysis through propensity scores-based matching. Cases were matched to controls according to the predicted probability of a diagnosis of CS. The matched cases and controls would be similar for all covariates used to calculate the propensity score [20]. Excluded from both cohorts were individuals younger than 18 years of age, those with a history of arrhythmia (ICD-9-CM code 427) at baseline, or those with no accompanying information relating to birth date and sex. The propensity score was calculated through logistic regression to estimate the probability of disease status, given the baseline variables, namely age; sex; frequency of medical visits/per year; occupation; urbanization level; comorbidities of diabetes, hypertension, hyperlipidemia, coronary artery disease (CAD), stroke, chronic kidney disease (CKD) or end-stage renal disease (ESRD), chronic obstructive pulmonary disease (COPD), cancer, congestive heart failure (CHF), and sleep apnea; and taking nonsteroidal anti-inflammatory drugs (NSAID), beta blockers, propranolol, carvedilol, and bisoprolol as medication. The comorbidities were defined prior to index CS diagnosis. Comorbidities were considered while they had received medical care at least three times outpatient visits and/or one-time hospitalizations. Most of the administrative cods of comorbidities used in this study have been reported for studies [21,22,23].

### 2.4. Outcomes

Both the CS and non-CS participants were followed until 31 December 2011, except when one of the following was reached first: the endpoint of arrhythmia was diagnosed, participant data was censored because of loss to follow-up, or participant withdrew from insurance program. The possible reasons for withdrawal from national health insurance include death, withdrawal of insurance, immigration, prison sentence, etc.

### 2.5. Statistical Analysis

The standardized mean difference was used to examine differences between the case group and the control group for categorical and continuous variables [24]. The cumulative incidence of arrhythmia between the two cohorts was plotted through the Kaplan-Meier method [25], and the difference was tested using the log-rank test. Incidence density rates of arrhythmia were also calculated for the case group, associated subgroups, and the control group by sex, age, and comorbidities. Cox proportional hazards regression analysis [26] was used to assess the hazard ratio (HR), and a 95% confidence interval (CI) of arrhythmia was associated with the case group relative to the control group. A multivariable model was used to estimate the adjusted HR (aHR), controlling for covariates that were significant in the univariable model [26]. The data was then analyzed to assess whether the treatment received or the type of CS had a role in arrhythmia outcomes. All statistical analyses were performed using SAS 9.3 software (SAS Institute, Cary, NC, USA) for Windows, and the level of significance was set at 0.05 using a two-tailed test.

## 3. Results

The case group comprised 22,236 subjects with CS and the control group comprised 22,236 subjects (Table 1).

**Figure 1:**
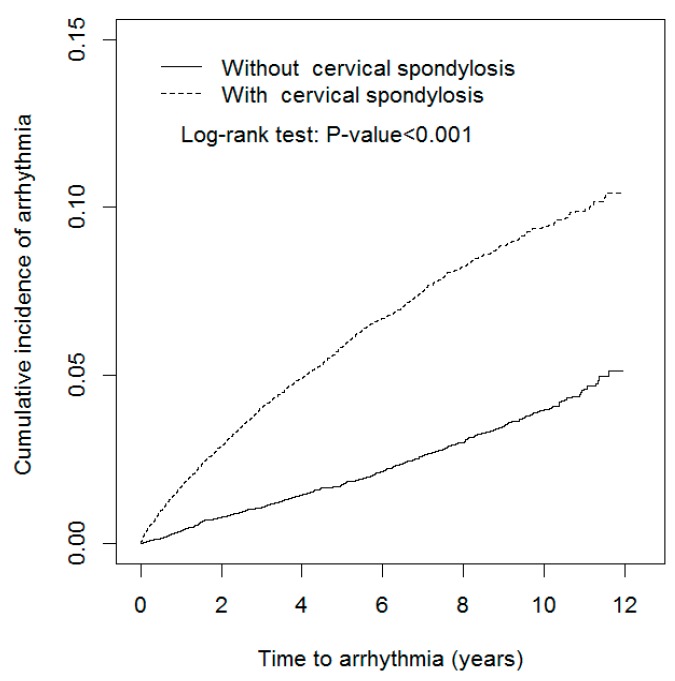
Cumulative incidence of arrhythmia in propensity-score matched patients with and without cervical spondylosis.

There were more women and younger individuals (mean age of 54.3 (SD 13.3) years) in the case group than in the control group (mean age of 54.4 (SD 13.4) years). The mean frequency of medical visits was higher in the case group (15.8 ± 13.9 medical visits per year) than in the control group (15.1 ± 13.9 medical visits per year). Nearly 50% of subjects worked in a white-collar profession (51.4% vs. 51.3% in the case and control groups, respectively) and most individuals lived in an urbanized area (63.4% vs. 63.6% in the case and control groups, respectively). The major comorbidity in both cohorts was hypertension (38.1% vs. 38.6%), followed by hyperlipidemia (30.5% vs. 31.0%), and COPD (13.7% vs. 13.9%). The percentage of patients taking NSAIDs was 34.3% vs. 34.4%. The mean follow-up period was shorter in the case group (5.86 ± 3.26 years) than in the control group (6.18 ± 3.15 years). The Kaplan–Meier plot revealed that the cumulative incidence of arrhythmia in the case group was approximately 5.44% higher than in the control group (log-rank test *p* < 0.001, Figure 1) after the 12-year follow-up.

The overall incidence density rates of arrhythmia were 11.1 and 3.91 per 1,000 person-years in the case and control groups, respectively (Table 2). The adjusted HRs (aHRs) of arrhythmia were 3.10 (95% CI = 2.80–3.42) in the case group relative to the control group, after controlling for age, sex, frequency of medical visits/per year, occupation, urbanization level, comorbidity of diabetes, hypertension, hyperlipidemia, CAD, stroke, CKD or ESRD, COPD, cancer, CHF, and medications of NSAID, beta blocker, propranolol, carvedilol, and bisoprolol. The risk of arrhythmia increased with age, with aHRs of 1.86 (95% CI = 1.64–2.11) and 2.68 (95% CI = 2.33–3.10) for the two older age groups. Compared with patients who worked in a white-collar profession, those with a blue-collar profession (aHR = 1.11, 95% CI = 1.00–1.23) and those working in other professions (aHR = 1.22, 95% CI = 1.07–1.39) had a higher risk of developing arrhythmia. The risk of developing arrhythmia was higher for individuals with hypertension (aHR = 1.75, 95% CI = 1.55–1.97), hyperlipidemia (aHR = 1.44, 95% CI = 1.30–1.59), CAD (aHR = 1.94, 95% CI = 1.74–2.17), COPD (aHR = 1.42, 95% CI = 1.27–1.60), and CHF (aHR = 1.24, 95%CI = 1.00–1.54).

The incidence densities of arrhythmia, as stratified by sex, age, occupation category, urbanization level, and comorbidities, were all higher in the case group than in the control group (Table 3). The aHRs of arrhythmia for the case group, as compared with the control group, were all significant for women, highest in patients aged ≤49 years, urbanization level 3, and patients with no comorbidities.

Further analysis revealed overall incidence density rates of arrhythmia of 10.8 and 11.5 per 1000 person-years for those in the case group without myelopathy and those in the case group with myelopathy, respectively, with aHRs of 2.87 (95% CI = 2.58–3.20) and 3.53 (95% CI = 3.13–3.97), respectively (Table 4).

Table 5 shows the relative risk of an individual in the case group developing arrhythmia after receiving treatment and not receiving treatment, respectively. Compared with the control group, participants in the case group with no neurological signs had a 3.32-fold increased risk of developing arrhythmia (95% CI = 3.01–3.68), whereas those receiving rehabilitation and with neurological signs receiving spinal decompression had a 1.94-fold and 1.84-fold increased risk of arrhythmia, respectively. Compared to patients in the case group with no neurological signs, those in the case group receiving rehabilitation had a 0.61-fold decreased risk of developing arrhythmia (95% CI = 0.48–0.78), whereas those with neurological signs receiving spinal decompression exhibited had a 0.58-fold decreased risk of developing arrhythmia (95% CI = 0.42–0.79).

Table 6 shows the relative risks for different types of arrhythmias in the case group. There were 252 events of atrial fibrillation in CS cohort and 127 events of atrial fibrillation in non-CS cohort, 33 events of ventricular tachycardia in CS cohort and 12 events of ventricular tachycardia in non-CS cohort, 78 events of supraventricular tachycardia in CS cohort and 35 events of supraventricular tachycardia in non-CS cohort. Compared with the control group, subjects in the case group had 2.22-fold increased risk of developing atrial fibrillation (95% CI = 1.79–2.76), a 3.19-fold increased risk of developing ventricular tachycardia (95% CI = 1. 64–6.20), and a 2.54-fold increased risk of developing supraventricular tachycardia (95% CI = 1.70–3.79).

## 4. Discussion

The present study reveals that CS is associated with a 3.10-fold increase of arrhythmia risk compared to patients without cervical spondylosis, especially atrial fibrillation, ventricular and supraventricular tachycardia. ANS stimulation could explain the related mechanism of this finding. Clinical evidence had shown that CS can cause sympathetic nerve irritation and associated sympathetic symptoms [27], and instability at the C4–C5 intervertebral space is the most-common type causing sympathetic symptoms [28]. Recognized sympathetic symptoms of CS are vertigo, dizziness, tinnitus, headache, and palpitation [29]. Previous electrophysiological studies have demonstrated that sympathetic activation has a proarrhythmic effect in enhancing automaticity, triggering re-entry, and reducing the threshold of refractoriness [29,30,31]. Such studies have shown that an imbalance of the autonomic nervous system and stimulation of the sympathetic nervous system can trigger atrial fibrillation [32], ventricular tachycardia [33], and ventricular fibrillation [34]. Such findings are thus similar to those of our study: CS is associated with a higher risk of developing atrial fibrillation, atrial flutter, and ventricular tachycardia. We supposed that a possible mechanism would be that cervical region trauma influences spinal sympathetic neurons because sympathetic control of heart originates from T1-5, thus the parasympathetic tone is unopposed. The increased vagal tone would be associated with atrial fibrillation. A reason for the insignificant risk of developing ventricular fibrillation shown in our study is possibly related to low event rate which only one event occurred in the study cohort and comparison cohort individually.

Another possible pathway is regional hyper innervation of the sympathetic nerves of the heart [35]. Although an increased nerve fiber density, and thus increased arrhythmia susceptibility, are most commonly seen in heart failure [36] and acute myocardial infarction [37], injury of the spinal cord is also one of the causes of hyper innervation [38]. We therefore hypothesize that CS could contribute to the possible irritation and injury of the spinal cord, which therefore causes hyper innervation of the heart. Electrophysiological evidence is therefore required to clarify the possible link between CS and hyper innervation of the sympathetic nerves. Our data also showed that beta-blockers bisoprolol and carvedilol was associated with lower risk of arrhythmia, while propranolol is associated with higher risk of arrythmia. Although these medications were all beta-receptor antagonists, we suppose that the difference would be that propranolol is widely used for anxiety beyond the range of cardiovascular system and its duration is short-acting [39,40]. Bisoprolol is a selective beta-1 receptor antagonist and carvedilol is a comprehensive beta (1)-, beta (2)-, and alpha (1)-adrenoreceptor blocker [41,42]. The lower risk of arrhythmia in users of bisoprolol and carvedilol might strengthen our supposed commonality mechanism that higher risk of arrhythmia cervical spondylosis patients is associated with hyperactive sympathetic tones in cervical spondylosis patients.

One interesting finding of this study is that CS patients who had received rehabilitation or surgical decompression had a lower risk of arrhythmia events compared with who had mild cervical spondylosis with no signs. A possible reason for this is that patients receiving surgical decompression were in relatively better physical health than those not receiving such an intervention; however, this is unlikely in this study as the prevalence of all possible comorbidities were comparable between the case group and the control group. Nevertheless, this study provides evidence supporting the use of rehabilitation and surgical decompression to protect against future arrhythmias, and the use of a detailed animal model is suggested to clarify the decompression effect on the cervical spinal cord with respect to arrhythmia.

This study has several limitations: first, information relating to smoking habits, alcohol consumption, caffeine intake, body mass index, and physical activity were unavailable from the NHIRD. Second, several confounders that could also cause arrhythmia, such as heart surgery, thoracic surgery, congenital heart anomaly, valvar heart disease, prolongation of QT interval, and thyroid disease were not considered in this study; this structural heart disease would be arrhythmogenic [43,44]. However, they were not frequently observed in people with cervical spondylosis, then this is less of a concern. Several other ECG markers of arrhythmia risk such as *P* wave duration, QRS duration and fragmentation, ST segment depression and elevation, Tpeak-Tend interval or premature ventricular contractions were also insufficient. Though other major comorbidities that could predispose to the development of arrhythmia were adjusted and matched to alleviate the bias, their associated therapy might influence arrhythmia risk were not considered. Third, the occurrence of arrhythmia was based on the diagnostic code registered in NHIRD; therefore, validation of an accurate arrhythmia diagnosis is lacking. Fourth, CS is common in the population and is likely to be underdiagnosed; therefore, a bias for CS prevalence is present in our case and control groups. Nevertheless, this bias is common in population-based studies, and we have included intervention procedures used for CS, such as surgical decompression and rehabilitation, to reinforce our findings. Furthermore, this study is retrospective-based and if subjects in the control group had undiagnosed CS, the risk of arrhythmia would therefore be underestimated in the case group, which thus strengthens the findings of this study: the risk of arrhythmia is increased in the CS population. Fifth, although we have considered occupation, we have no information about night shift conditions of each participant. Night shift is reported to be associated with prolong QT and Tpeak-Tend intervals, which increases ventricular arrhythmia risk [45]. Finally, the different types of arrhythmia, such as atrial fibrillation, atrioventricular node block, QT prolongation, and sick sinus syndrome, were not differentiated in this study. Nevertheless, because the objective of this study was to evaluate the association between CS and the risk of developing arrhythmia, the limitations presented here do not affect the value of the findings.

In summary, this study demonstrated that CS is associated with an increased risk of developing arrhythmia. As arrhythmia is linked to the risk of sudden death and strokes, this study should prompt clinical awareness of the higher risk of arrhythmia in patients with CS. The finding of this study might arouse the alertness of clinicians that CS is associated with arrhythmia, especially those who had symptoms of myelopathy. Thus, electrocardiogram would be needed in those CS presented with sympathetic symptoms or those who had several identified risk markers of arrhythmia. Primary prevention with anti-coagulations or anti-arrhythmic medications for fatal arrhythmia or ischemia stroke would be beneficial. This study has several inherent limitations; thus, large prospective studies are required to examine the direct causal relationship between CS and the occurrence of arrhythmia.

## Figures and Tables

**Table 1:** Demographic characteristics and comorbidities of propensity-score matched patients with and without cervical spondylosis.

	Cervical Spondylosis		Standard Mean Difference ^§^
	No	Yes	
	(*N* = 22,236)	(*N* = 22,236)	
Sex			
Women	12,791 (57.5)	12,766 (57.4)	0.002
Men	9445 (42.5)	8470 (42.6)	0.002
Age stratified			
≤49	8067 (36.3)	8817 (39.7)	0.07
50–64	8885 (40.0)	8407 (37.8)	0.04
65 +	5284 (23.8)	5012 (22.5)	0.03
Age, mean ± SD	54.4 (13.4)	54.3 (13.3)	0.01
Frequency of medical visits/per year, Means (SD) ^†^	15.1 (13.9)	15.8 (13.9)	0.05
Occupation			
White collar	11,412 (51.3)	11,429 (51.4)	0.002
Blue collar	7889 (35.5)	7884 (35.5)	0.000
Others ^&^	2935 (13.2)	2923 (13.2)	0.002
Urbanization level ^†^			
1 (highest)	7353 (33.1)	7373 (33.2)	0.002
2	6782 (30.5)	6723 (30.2)	0.01
3	3534 (15.9)	3594 (16.2)	0.01
4 (lowest)	4567 (20.5)	4546 (20.4)	0.002
Comorbidity			
Diabetes	2385 (10.7)	2352 (10.6)	0.005
Hypertension	8574 (38.6)	8471 (38.1)	0.01
Hyperlipidemia	6887 (31.0)	6785 (30.5)	0.01
CAD	4366 (19.6)	4276 (19.2)	0.01
Stroke	903 (4.06)	898 (4.04)	0.001
CKD or ESRD	338 (1.52)	329 (1.48)	0.003
COPD	3093 (13.9)	3054 (13.7)	0.01
Cancer	623 (2.80)	618 (2.78)	0.001
CHF	560 (2.52)	533 (2.40)	0.01
Sleep apnea	114 (0.51)	114 (0.51)	0.000
Medications			
NSAID	7649 (34.4)	7627 (34.3)	0.002
All Beta blocker	9088 (40.9)	9062 (40.8)	0.002
Propranolol	6293 (28.3)	6330 (28.5)	0.004
Carvedilol	789 (3.55)	782 (3.52)	0.002
Bisoprolol	1355 (6.09)	1348 (6.06)	0.001

^§^ A standardized mean difference of ≤0.10 indicates a negligible difference between the two cohorts. ^†^ The urbanization level was categorized by the population density of the residential area into 4 levels, with level 1 as the most urbanized and level 4 as the least. ^&^ Other occupation categories included those who were primarily retired, unemployed, and low-income populations.

**Table 2:** Incidence and Hazard ratio for Arrhythmia and Arrhythmia-associated risk factor.

Variable	Event	PY	Rate ^#^	Crude HR (95% CI)	Adjusted HR ^§^ (95% CI)
Cervical spondylosis					
No	537	137,401	3.91	1.00	1.00
Yes	1441	130,269	11.1	2.82 (2.55, 3.11) ***	3.10 (2.80, 3.42) ***
Age group, year					
20–49	440	108,197	4.07	1.00	1.00
50–64	787	102,519	7.68	1.87 (1.66, 2.10) ***	1.86 (1.64, 2.11) ***
≥65	751	56,953	13.2	3.19 (2.83, 3.59) ***	2.68 (2.33, 3.10) ***
Sex					
Female	1134	158,588	7.15	1.00	1.00
Male	844	109,082	7.74	1.07 (0.98, 1.17)	
Occupation					
White collar	887	135,455	6.55	1.00	1.00
Blue collar	761	96,808	7.86	1.21 (1.10, 1.33) ***	1.11 (1.00, 1.23) *
Others ^&^	330	35,407	9.32	1.43 (1.26, 1.62) ***	1.22 (1.07, 1.39) **
Urbanization level ^†^					
1 (highest)	615	88,016	6.99	1.00	1.00
2	584	81,759	7.14	1.02 (0.91, 1.15)	1.01 (0.90, 1.14)
3	313	42,595	7.35	1.05 (0.92, 1.21)	1.05 (0.92, 1.20)
4(lowest)	466	55,300	8.43	1.21 (1.07, 1.36) **	1.07 (0.94, 1.22)
Comorbidity					
Diabetes					
No	1756	241,894	7.26	1.00	1.00
Yes	222	25,776	8.61	1.17 (1.01, 1.34) *	0.86 (0.75, 1.00)
Hypertension					
No	877	169,015	5.19	1.00	1.00
Yes	1101	98,655	11.2	2.13 (1.95, 2.33) ***	1.75 (1.55, 1.97) ***
Hyperlipidemia					
No	1200	188,751	6.36	1.00	1.00
Yes	778	78,919	9.86	1.54 (1.40, 1.68) ***	1.44 (1.30, 1.59) ***
CAD					
No	1261	218,556	5.77	1.00	1.00
Yes	717	49,114	14.6	2.51 (2.29, 2.75) ***	1.94 (1.74, 2.17) ***
Stroke					
No	1885	258,451	7.29	1.00	1.00
Yes	93	9219	10.1	1.35 (1.10, 1.67) **	0.72 (0.58, 0.89) **
CKD or ESRD					
No	1939	264,481	7.33	1.00	1.00
Yes	39	3189	12.2	1.61 (1.17, 2.21) **	0.93 (0.67, 1.29)
COPD					
No	1569	233,782	6.71	1.00	1.00
Yes	409	33,888	12.1	1.77 (1.59, 1.98) ***	1.42 (1.27, 1.60) ***
Cancer					
No	1917	261,697	7.33	1.00	1.00
Yes	61	5973	10.2	1.36 (1.05, 1.75) *	1.18 (0.91, 1.53)
CHF					
No	1878	262,212	7.16	1.00	1.00
Yes	100	5458	18.3	2.49 (2.04, 3.04) ***	1.24 (1.00, 1.54) *
Sleep apnea					
No	1968	266,718	7.38	1.00	1.00
Yes	10	952	10.5	1.34 (0.72, 2.49)	
Medication					
NSAID					
No	1003	136,500	7.35	1.00	1.00
Yes	975	131,170	7.43	1.12 (1.02, 1.23) *	2.49 (2.25, 2.75) ***
All Beta blocker					
No	915	165,352	5.53	1.00	1.00
Yes	1063	102,318	10.4	1.85 (1.69, 2.02) ***	1.20 (1.03, 1.39) *
Propranolol					
No	1227	197,835	6.20	1.00	1.00
Yes	751	69,835	10.8	1.71 (1.56, 1.87) ***	1.43 (1.25, 1.64) ***
Carvedilol					
No	1886	260,263	7.25	1.00	1.00
Yes	92	7407	12.4	1.65 (1.34, 2.04) ***	0.79 (0.64, 0.99) *
Bisoprolol					
No	1830	255,203	7.17	1.00	1.00
Yes	148	12,467	11.9	1.59 (1.34, 1.88) ***	0.74 (0.62, 0.89) **

CI, confidence interval; COPD, chronic obstructive pulmonary disease; CKD, chronic kidney disease; HR, hazard ratio; PY, person-years; ^#^ Incidence rate per 1000 person-years; ^§^ Model was adjusted for age, sex, frequency of medical visits/per year, occupation, urbanization level, comorbidity of diabetes, hypertension, hyperlipidemia, CAD, stroke, CKD or ESRD, COPD, cancer, CHF, and medications of NSAID, beta blocker, propranolol, carvedilol, and bisoprolol by using Cox proportional hazards regression; ^†^ The urbanization level was categorized by the population density of the residential area into 4 levels, with level 1 as the most urbanized and level 4 as the least urbanized. ^&^ Other occupation categories included those who were primarily retired, unemployed, and low-income populations. * *p* < 0.05, ** *p* < 0.01, *** *p* < 0.001.

**Table 3:** Comparison of incidence densities of Arrhythmia and Cox model measured hazard ratio between patients with and without cervical spondylosis by demographic characteristics and comorbidity.

	Cervical Spondylosis							
	No			Yes				
	Event	PY	Rate ^#^	Event	PY	Rate ^#^	Crude HR (95% CI)	Adjusted HR ^§^ (95% CI)
Sex								
Women	293	81,923	3.58	841	76,664	11.0	3.04 (2.66, 3.48) ***	3.38 (2.95, 3.86) ***
Men	244	55,477	4.40	600	53,605	11.2	2.54 (2.19, 2.94) ***	2.84 (2.44, 3.30) ***
Stratify age								
≤49	99	51,648	1.92	341	56,550	6.03	3.14 (2.51, 3.93) ***	3.61 (2.88, 4.53) ***
50–64	213	54,428	3.91	574	48,091	11.9	3.03 (2.59, 3.55) ***	3.48 (2.97, 4.08) ***
65+	225	31,325	7.18	526	25,628	20.5	2.81 (2.40, 3.29) ***	2.77 (2.37, 3.25) ***
Occupation								
White collar	252	68,498	3.68	635	66,957	9.48	2.57 (2.22, 2.98) ***	2.98 (2.57, 3.45) ***
Blue collar	209	50,511	4.14	552	46,298	11.9	2.85 (2.43, 3.35) ***	2.98 (2.57, 3.45) ***
Others ^&^	76	18,392	4.13	254	17,015	14.9	3.59 (2.78, 4.63) ***	3.63 (2.80, 4.70) ***
Urbanization level ^†^								
1 (highest)	176	44,789	3.93	439	43,227	10.2	2.57 (2.16, 3.06) ***	2.91 (2.44, 3.47) ***
2	166	41,892	3.96	418	39,867	10.5	2.64 (2.20, 3.16) ***	2.95 (2.46, 3.53) ***
3	66	21,780	3.03	247	20,815	11.9	3.89 (2.96, 5.10) ***	4.23 (3.22, 5.56) ***
4 (lowest)	129	28,940	4.46	337	26,360	12.8	2.84 (2.32, 3.48) ***	3.01 (2.45, 3.69) ***
Comorbidity ^‡^								
No	115	60,271	1.91	344	59,401	5.79	3.04 (2.46, 3.75) ***	3.66 (2.96, 4.53) ***
Yes	422	77,130	5.47	1097	70,868	15.5	2.80 (2.50, 3.13) ***	2.89 (2.58, 3.23) ***

Rate ^#^, incidence rate, per 1000 person-years; Crude HR, relative hazard ratio; Adjusted HR ^§^: mutually adjusted for age, sex, frequency of medical visits/per year, occupation, urbanization level, comorbidity of diabetes, hypertension, hyperlipidemia, CAD, stroke, CKD or ESRD, COPD, cancer, CHF, and medications of NSAID, beta blocker, propranolol, carvedilol, and bisoprolol by using Cox proportional hazards regression; Comorbidity ^‡^: Patients with any one of the comorbidities diabetes, hypertension, hyperlipidemia, CAD, stroke, CKD or ESRD, COPD, cancer, CHF, sleep apnea were classified as the comorbidity group. ^†^ The urbanization level was categorized by the population density of the residential area into 4 levels, with level 1 as the most urbanized and level 4 as the least urbanized. ^&^ Other occupation categories included those who were primarily retired, unemployed, and low-income populations. *** *p* < 0.001.

**Table 4:** Incidences and hazard ratios of arrhythmia in cervical spondylosis patients with and without myelopathy compared to patients without cervical spondylosis.

Variable	N	Events	PYs	Rate ^#^	Crude HR (95% CI)	Adjusted HR ^&^ (95% CI)
Without cervical spondylosis	22,236	537	137,401	3.91	1.00	1.00
Type of Cervical spondylosis						
Cervical spondylosis without myelopathy	14,414	882	81,743	10.8	2.73 (2.46, 3.04) ***	2.87 (2.58, 3.20) ***
Cervical spondylosis with myelopathy	7822	559	48,526	11.5	2.95 (2.62, 3.33) ***	3.53 (3.13, 3.97) ***

Rate ^#^, incidence rate, per 1000 person-years; Crude HR, relative hazard ratio; Adjusted HR ^&^: mutually adjusted for age, sex, frequency of medical visits/per year, occupation, urbanization level, comorbidity of diabetes, hypertension, hyperlipidemia, CAD, stroke, CKD or ESRD, COPD, cancer, CHF, and medications of NSAID, beta blocker, propranolol, carvedilol, and bisoprolol by using Cox proportional hazards regression; *** *p* < 0.001.

**Table 5:** Incidences and hazard ratios of arrhythmia for cervical spondylosis patients with and without treatment compared to patients without cervical spondylosis.

Variable	N	Events	PYs	Rate ^#^	Crude HR (95% CI)	Adjusted HR ^&^ (95% CI)	Crude HR (95% CI)	Adjusted HR ^&^ (95% CI)
Without cervical spondylosis	22,236	537	137,401	3.91	1.00	1.00		
With cervical spondylosis								
Mild: cervical spondylosis no signs	19,047	1335	107,982	12.4	3.13 (2.84, 3.47) ***	3.32 (3.01, 3.68) ***	1.00	1.00
Moderate: cervical spondylosis receiving rehabilitation	1996	67	14,352	4.67	1.22 (0.94, 1.57)	1.94 (1.51, 2.51) ***	0.40 (0.31, 0.50) ***	0.61 (0.48, 0.78) ***
Severe: cervical spondylosis with neurological signs receiving spinal decompression	1193	39	7935	4.91	1.27 (0.92, 1.75)	1.84 (1.33, 2.55) ***	0.41 (0.30, 0.56) ***	0.58 (0.42, 0.79) ***

Rate ^#^, incidence rate, per 1000 person-years; Crude HR, relative hazard ratio; Adjusted HR ^&^: mutually adjusted for age, sex, frequency of medical visits/per year, occupation, urbanization level, comorbidity of diabetes, hypertension, hyperlipidemia, CAD, stroke, CKD or ESRD, COPD, cancer, CHF, and medications of NSAID, beta blocker, propranolol, carvedilol, and bisoprolol by using Cox proportional hazards regression; *** *p* < 0.001.

**Table 6:** Comparison of incidence densities of different types of arrhythmia and Cox model measured hazard ratio between patients with and without cervical spondylosis.

	Cervical Spondylosis					
	No		Yes			
Variable (ICD-9-CM)	Event	Rate ^#^	Event	Rate ^#^	Crude HR (95% CI)	Adjusted HR ^§^ (95% CI)
Atrial fibrillation (427.31)	127	0.92	252	1.93	2.08 (1.68, 2.58) ***	2.22 (1.79, 2.76) ***
Ventricular tachycardia (427.1)	12	0.09	33	0.25	2.89 (1.49, 5.59) **	3.19 (1.64, 6.20) ***
Supraventricular tachycardia (427.0)	35	0.25	78	0.60	2.33 (1.56, 3.47) ***	2.54 (1.70, 3.79) ***

Rate ^#^, incidence rate, per 1000 person-years; Crude HR, relative hazard ratio; Adjusted HR ^§^: mutually adjusted for age, sex, frequency of medical visits/per year, occupation, urbanization level, comorbidity of diabetes, hypertension, hyperlipidemia, CAD, stroke, CKD or ESRD, COPD, cancer, CHF, and medications of NSAID, beta blocker, propranolol, carvedilol, and bisoprolol by using Cox proportional hazards regression; Due to low event rates, atrial flutter and ventricular fibrillation were not presented. ** *p* < 0.01, *** *p* < 0.001.
